# Evaluation of ground based spectral imaging for real time maize biomass monitoring

**DOI:** 10.3389/fpls.2025.1566305

**Published:** 2025-06-13

**Authors:** Andrea Szabó, Nxumalo Gift Siphiwe, Erika Buday-Bódi, Blessing Ademola, János Tamás, Attila Nagy

**Affiliations:** ^1^ Institute of Water and Environmental Management, Faculty of Agricultural and Food Sciences and Environmental Management, University of Debrecen, Debrecen, Hungary; ^2^ National Laboratory for Water Science and Water Safety, Institute of Water and Environmental Management, Faculty of Agricultural and Food Sciences and Environmental Management, University of Debrecen, Debrecen, Hungary; ^3^ Department of Biological Engineering, University of Missouri, Columbia, MO, United States

**Keywords:** NDVI, linear regression, biomass, multispectral imaging, maize

## Abstract

Although point measurements of water management properties have become increasingly common, understanding the spatial heterogeneity of agricultural fields remains critical for advancing precision agriculture. Spectral analysis provides a non-destructive approach to evaluating plant biophysical properties, such as chlorophyll and carotenoids, which are critical for precision agriculture. This study addresses the challenge of precise plant trait prediction by integrating proximal sensing data with biomass observations to inform more effective water management strategies. This study predicts carotenoid and chlorophyll content from NDVI, and estimates dry and wet biomass from vegetation cover using multispectral Tetracam data. A key novel aspect of this study lies in the pioneering integration of proximal sensing with biomass information to improve the estimation of plant properties, offering practical applications for precision agriculture. The diagnostic results demonstrated varying model performances. The carotenoid prediction model, with a moderate R² (0.54), exhibited a slight overestimation, characterized by a Mean Bias Error (MBE) of 0.02 µg/g and a Normalized Root Mean Square Error (NRMSE) of 17%. Conversely, the chlorophyll prediction model showed improved accuracy, achieving an R² of 0.64, an MBE of 0.04 µg/g, and an NRMSE of 15.92%. Models predicting wet and dry biomass from vegetation cover yielded comparable performances, with R² values of 0.55 and 0.58, and low NRMSEs of 13.26% and 15.06%, respectively. These findings underscore the potential of combining proximal sensing and biomass data to enhance the prediction of plant properties, providing valuable insights for optimizing precision agriculture through machine learning.

## 1 Introduction

The increasing demand for water, intensified by climate change, calls for innovative solutions that promote sustainable and efficient agricultural practices. Precision irrigation, particularly Variable Rate Irrigation (VRI), addresses this by delivering water according to crop needs using spatial data on soil, crop type, and topography (Yari et al., 2017; O’Shaughnessy et al., 2019). Real-time monitoring of crop conditions and water requirements, often through soil sensors or evapotranspiration models, is critical for optimizing irrigation (Siphiwe et al., 2024). Despite challenges in defining stress thresholds, plant-based indicators like the Plant Water Stress Index show promise (Alharbi et al., 2024).

Traditional biomass estimation methods are labor-intensive and limited in scale, leading to increased use of satellite, Unmanned Aerial Vehicle (UAV), and ground-based sensors. Vegetation indices derived from spectral reflectance data (e.g., Normalized Difference Vegetation Index (NDVI)) offer reliable insights into plant health and biomass (Nigon et al., 2014; Morier et al., 2015). Studies confirm strong correlations between spectral data and biochemical properties like chlorophyll and carotenoids (Ranjan and Parida, 2020; Pandey et al., 2023), with satellite data proving effective for biomass estimation (Marcone et al., 2024; Nagy and Tamás, 2013).

Combining ground-based sensing with aerial or satellite platforms enhances resolution and coverage (Pettorelli, 2019), though challenges in data integration, calibration, and resolution alignment remain (Zhu et al., 2018).Techniques like Principal Component Analysis (PCA) help identify key variables—NDVI, chlorophyll, and carotenoids—as indicators of crop health (Todorova et al., 2024).

Ground-based sensors provide detailed spectral data for applications such as crop row detection, irrigation management, and small-area monitoring (Caturegli et al., 2016; Ronchetti et al., 2020). They also improve calibration of remote sensing data and support biomass estimation (Ahmad et al., 2022; Rehman and Lundy, 2022). These sensors have been used for Leaf Area Index (LAI) estimation, water stress detection, disease diagnosis, and nutrient analysis in various crops (Towers et al., 2019; Zhou et al., 2022; Liu et al., 2024). Recent innovations in biomass monitoring have emphasized the need for improved spectral fidelity and environmental robustness in sensing technologies. For instance Xie et al. (2024), demonstrated that proximal sensing platforms significantly reduce atmospheric spectral distortions compared to UAV-based systems, while (Manley et al., 2025) showed that integrating real-time white references and shadow correction techniques enhances spectral accuracy under variable light conditions.

However, limitations include high cost, limited spatial coverage, and challenges with real-time processing—particularly with hyperspectral sensors, which require significant computational resources and are affected by environmental conditions (Bioucas-Dias et al., 2013; Omia et al., 2023). Moreover, biomass models often lack generalizability across conditions (Sileshi, 2014), and plant density significantly influences biomass traits (Poley and McDermid, 2020; Shah et al., 2021).

As a staple crop, maize requires accurate, timely monitoring to address stress and nutrient deficiencies. Proximal sensing offers a practical solution for in-season assessments of growth and biomass, helping optimize water and nutrient use (Laveglia et al., 2024).

The novel contribution of this ground-based multispectral imaging study goes beyond cost-effectiveness, aiming to assess whether the system offers higher spatial and temporal resolution, reduces atmospheric interference, and enables flexible deployment under variable weather conditions (Alexopoulos et al., 2023). The study aims to demonstrate how spectral imaging can improve irrigation and nutrient management, enhancing yield and sustainability in maize production. The aim of this study was to assess the feasibility of using spectral data captured by an imaging sensor to evaluate biomass in maize fields. The specific objectives were as follows: (1) To collect spectral data using a multispectral camera, enabling the calculation of NDVI and the quantification of vegetation cover; (2) To characterize the spectral properties of maize fields and carry out calibration and validation procedures for accurate analysis; (3) To apply linear regression to explore the relationship between NDVI and the chlorophyll and carotenoid content of maize, as well as to use vegetation cover data to estimate both wet and dry biomass. The main goal was to investigate how information obtained from spectral imaging can support the optimization of irrigation and nutrient management practices. The study integrated spectral data with crop monitoring to evaluate the effectiveness of proximal sensing technologies in real-time monitoring of biomass distribution, plant health, and nutritional status. This approach could contribute to more precise and resource-efficient management of water and fertilizers in maize cultivation, ultimately enhancing yield and promoting sustainability.

## 2 Materials and methods

### 2.1 Study site

The research site comprised a 53-hectare irrigated maize field located in the Pannonian region northeastern part of Hungary, situated on the border of a climate belt that is both moderately warm and cool. This case study area (latitude: 47°48’18.60”N, longitude: 22°9’43.89”E, altitude: 144 m) owned by a private company, falls within a nitrate-sensitive zone. The topography of the site, located on an alluvial cone plain, predominantly consists of quicksand, posing suboptimal conditions for maize production, particularly in relation to water management. Maize holds significant agricultural importance both in Europe and Hungary. The maize (*KWS Kleopatras* variety) was planted on early May, 2021, with a seeding rate of 72,000 seeds per hectare, and the harvest took place on mid-September. In 2022, the PIONEER P0725 maize variety was used for sowing. 4644.40 q silos were harvested on the irrigated area, resulting in an average yield of 344.03 q/ha. The main pests in the field were corn earworm, cotton-worm, and corn borer. Secondary fungal infestations such as powdery mildew, fusarium and Aspergillus species also occurred due to the damage caused by these fungi. The irrigated area produced 4835.40 q silage with an average yield of 352.03 q/ha. Among the pests, corn earworm and cotton bollworm dominated, while the diseases fusarium and yellow rust continued to be more prevalent.

**Figure 1:**
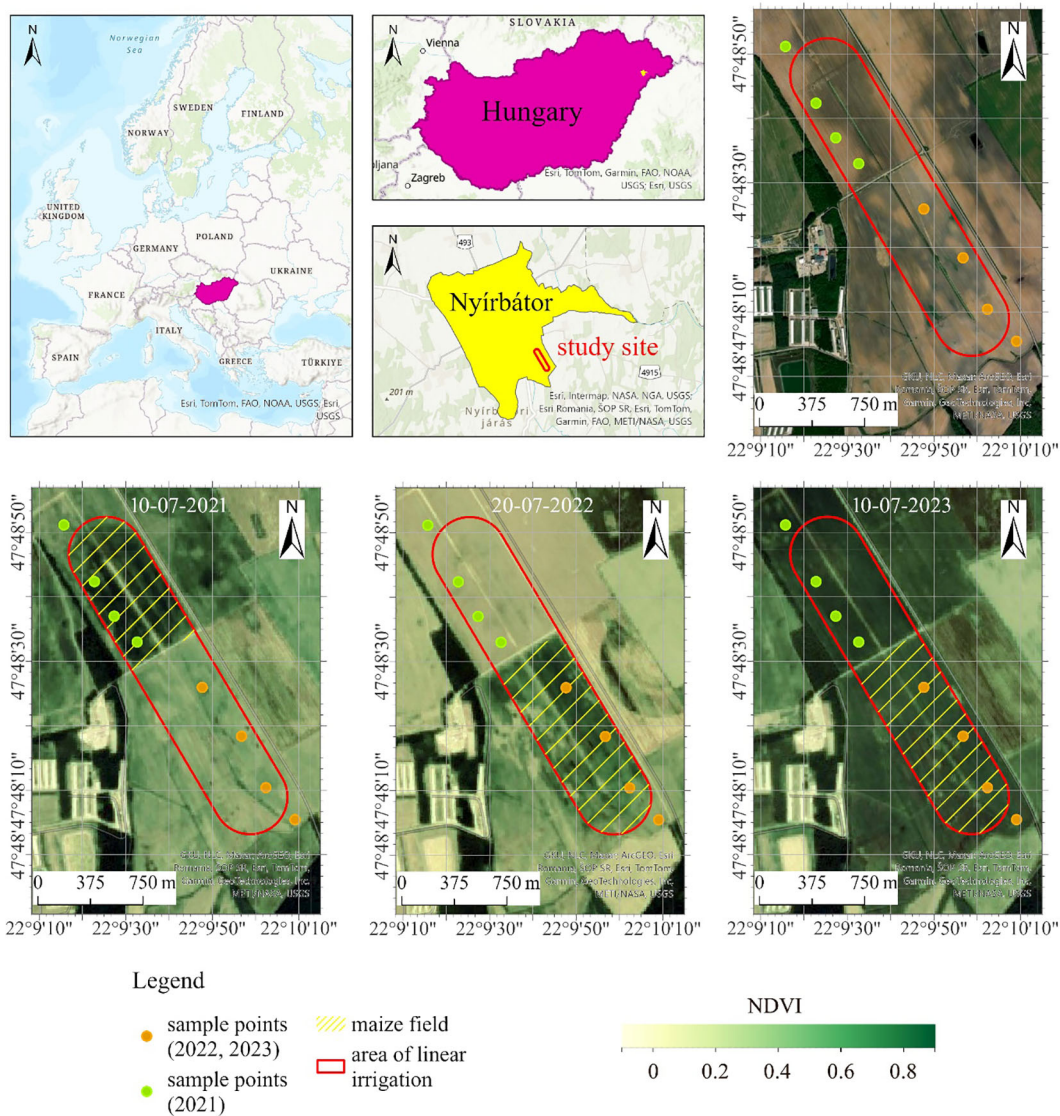
Study site with sample points.

Despite the total precipitation during the growing period reaching 304.4 mm, the field’s water demand for optimal growth is relatively high, ranging between 450–500 mm. According to Bencze (2022) to maize exhibits a daily water consumption rate of 4.5-5.5 mm ha^-1^ (45–55 m^3^ ha^-1^). Consequently, the study area was irrigated using a Reinke E2060PL pivoting linear irrigation machine equipped with VRI technology to achieve optimal water application (Szabó et al., 2023) (**Figure 1**).

### 2.2 Surveying the biomass

To explore the connection between NDVI and pigment contents, correlation analysis involving carotenoid content, chlorophyll, biomass weight, and spectral image-related data (NDVI, area of green biomass) was conducted. Maize plant samples were collected from heterogeneous patches within an 18 m wide by 150 m long irrigated plot during four sampling periods, as outlined in **Table 1**. The BBCH scale (Biologische Bundesanstalt, Bundessortenamt, and Chemical industry scale), a standardized system, is used to describe the phenological development stages of plants. Field surveys were conducted from early July to early September, encompassing four distinct BBCH growth stages: BBCH 30 (stem elongation), BBCH 50 (inflorescence emergence), BBCH 70 (milk development), and BBCH 80 (dough development). The plant age at the time of sampling ranged from approximately 30 to 70 days post-emergence, depending on the specific BBCH stage. This timeline ensured that data were collected across critical stages of vegetative and reproductive growth, providing a comprehensive understanding of pigment dynamics and biomass accumulation (Meier, 2001) (**Table 1**).

**Table 1:** Classification of maize according to the BBCH scale.

BBCH scale	2021	2022	2023
53 (Inflorescence emergence, heading - 30% of inflorescence emerged)	09. 07.	13. 06.	14. 06
69 (Flowering, anthesis - End of flowering: all spikelets have completed flowering, but some dehydrated anthers may remain	23. 07.	28. 06.	28. 07.
75 (Medium milk: grain content milky, grains reached final size, still green)	06. 08.	12. 08.	10. 08.
89 (Fully ripe: grain hard, difficult to divide with thumbnail)	02. 09.	17. 08.	31. 08


(1) $$NDVI=\frac{\text{NIR}+\text{RED}}{\text{NIR}-\text{RED}}\;\;\;$$

(Pelta et al., 2022)

Where:

NIR represents the near-infrared reflectance
RED represents the red reflectance

At each sampling time corresponding to various stages of maize development, three blocks, each measuring 5 × 5 meters, were carefully selected within the study area. These blocks were chosen to ensure representative coverage of the field conditions and variability. Within each block, four maize plants were systematically examined, providing a detailed dataset for analysis. The examination was conducted using a Tetracam ADC digital multispectral camera (Tetracam, Inc., CA, USA), a high-precision instrument designed to capture multispectral imagery across specific wavelengths. This imaging approach enabled the collection of detailed spectral data, which is crucial for assessing the physiological status and growth characteristics of the maize plants throughout their development. The structured sampling approach ensured consistency and reliability in the data collected across all developmental stages.

The camera captured images in the green (520–600 nm), red (620–750 nm), and near-infrared (750–950 nm) ranges. These wavelengths were selected based on their established relevance in capturing carotenoid and chlorophyll absorption peaks. The green range corresponds to carotenoid absorption, as carotenoids exhibit significant light absorption in this region. The red range is crucial for chlorophyll absorption, particularly around 640–680 nm, where chlorophyll-a and chlorophyll-b show maximum absorbance. The near-infrared range is sensitive to plant structure and biomass, providing critical information on vegetation health and density through reflectance characteristics (Samadzadegan et al., 2004). The surveys were conducted between 10:00 AM and 12:00 PM on the dates specified in **Table 1**. This time frame was chosen to ensure consistent lighting conditions and minimize the impact of varying light levels on plant pigments, as midday light provides stable and optimal conditions for accurate spectral data collection. A total of 48 plants were measured for calibration purposes, and an additional 30 plants were used for validation. Throughout the measurements, a white sheet was used to blank the background of the maize plants. The images of the maize foliage were consistently taken from the same side-look distance. NDVI (Equation 1) images were generated through image segmentation based on raw data using the PixelWrench2 software environment (Tetracam, Inc., CA, USA). From the NDVI images, histograms were extracted, providing NDVI values. Additionally, Boolean layers for vegetation areas were created through a straightforward classification of the NDVI image in ArcGIS. Utilizing the images, the area ratio of the observed plant was calculated, representing the ratio of vegetation-covered pixels to non-covered pixels (%) in the whole area. NDVI is widely used in agriculture due to its simplicity, cost-effectiveness, and non-invasive approach to assessing resource status (Vélez et al., 2023). The proposed Tetracam multispectral imaging approach enhances biomass estimation by reducing environmental noise through narrowband spectral filtering and radiometric correction (Roma et al., 2023). It also compensates for light variability using downwelling sensors and simultaneous band capture, ensuring consistent reflectance measurements under varying illumination (Haque et al., 2025). Additionally, built-in sensor calibration routines and standardized outputs enhance temporal and spatial consistency over traditional RGB or uncalibrated aerial systems (Xie et al., 2024a). Other devices may offer higher spectral resolution or additional features, but for the scope and objectives of this project, the Tetracam ADC provided the required performance at a manageable complexity and cost.

### 2.3 Measurement of pigment content and dry matter content

Concurrently, with the collection of spectral data, measurements of maize leaf pigment content were conducted. For each maize plant sample surveyed by the Tetracam ADC, 5 leaf samples were collected and readings taken across multiple stages (**Table 1**). The pigment results for each plant (48 plant samples) were calculated based on the mean of the 5 leaf samples from a specific plant. The estimation of pigment content accounted for potential effects of shading and leaf position by randomizing sample collection and standardizing measurement protocols.

The leaf samples’ pigment content was extracted by immersing and grinding the leaf sample in 80% acetone along with 1 g of quartz sand for homogeneity. After extraction, the suspensions underwent centrifugation at 3000 rev/min for 3 minutes, and the resulting clear solution was transferred to a 2.5 mL quartz cuvette. The absorbance of the solution was measured using a UV-VIS spectrophotometer at wavelengths of 470 nm, 644 nm, and 663 nm. The chlorophyll content of the samples was determined based on the equation published by (Droppa et al., 2003) (Equation 2). In the context of our study, ‘total chlorophyll’ refers to the combined concentration of chlorophyll-a and chlorophyll-b, the two primary pigments responsible for photosynthesis in plants. Total chlorophyll is relevant in our study as it is a critical indicator of plant health and productivity, it reflects the maize’s ability to capture light energy for photosynthesis. Accurately quantifying total chlorophyll provides valuable insights into the physiological status of the maize plants, enabling the assessment of their growth and biomass production under varying conditions.


(2) $$\begin{array}{c} Chlorophyll\;(a+b)(\mu g/g\;fresh\;weight)\\ =(20.2{*\text{A}}_{644\text{nm}}+8.02{*\text{A}}_{663\text{nm}})*\text{V}/\text{w} \end{array}$$

The carotenoid values were calculated based on Equation 3 (Lichtenthaler and Wellburn, 1983).


(3) $$\begin{array}{c} Carotenoid\;(\mu g\;/g\;fresh\;weight)=(1000*A_{470nm}\\ -3.27(12.21*A_{663nm}-2.81*A_{644nm})–104*\\ \left(20.13*A_{644nm}–5.03*A_{663nm}))/229*(V/w\right) \end{array}$$

Where:

V = volume of tissue extract (mL)
w = fresh mass of tissue (g^-1^)
A = absorbance to different (nm)

### 2.4 Statistical evaluation analysis

For the evaluation of pigment concentrations and TETRACAM ADC results, a statistical analysis was conducted using the SPSS software environment. This involved calculating general descriptive statistics, performing principal component analyses, and con-ducting correlation calculations. The Kaiser-Meyer-Olkin (KMO) test was employed to assess the suitability of principal component analyses, and Varimax rotation was applied. Correlations were examined among area ratio, mean NDVI data from the imaging procedure, as well as chlorophyll, carotenoid content, wet biomass, dry biomass, and dry matter content. Area ratio data represents the proportion of green plant biomass compared to the entire image. Firstly, the Pearson correlation coefficient was used to assess significant linear relationships (*P < 0.05 for moderate significance, *P < 0.01 for high significance), both positive and negative, between the predicted and observed biophysical data (Chicco et al., 2021; Obilor and Amadi, 2018). To assess the predictive models, performance metrics, including the coefficient of determination (R^2^), root mean square error (RMSE), and Normalized Root Mean Square Error (NRMSE), mean bias error (MBE), mean square error (MSE), and mean absolute error (MAE), were used (Chicco et al., 2021) (Equations 4–10). The evaluation was based on data from 26 sites (n = 30).


(4) $$r=\frac{n\sum_{i=1}^{n}(y_{i}\overset{´}{y_{i}})-\sum_{i=1}^{n}y_{i}\sum_{i=1}^{n}\overset{´}{y_{i}}}{\sqrt{\sum_{i=1}^{n}{\left(y_{i}-\bar{y}\right)}^{2}\sum_{i=1}^{n}{\left(y_{i}-\overset{´}{y_{i}}\right)}^{2}}}$$

(5) $$R^{2}=1-\frac{\sum_{i=1}^{n}{\left(y_{i}-\overset{´}{y_{i}}\right)}^{2}}{\sum_{i=1}^{n}{\left(y_{i}-\bar{y}\right)}^{2}}$$

(6) $$RMSE=\sqrt{\frac{\sum_{i=1}^{n}{\left(y_{i}-\overset{´}{y_{i}}\right)}^{2}}{n}}$$

(7) $$NRMSE=\frac{\sqrt{\frac{\sum_{i=1}^{n}{\left(y_{i}-\overset{´}{y_{i}}\right)}^{2}}{n}}}{\left(\bar{y}\right)}$$

(8) $$\;MSE=\frac{1}{n}\sum_{i=1}^{n}{\left(y_{i}-\overset{´}{y_{i}}\right)}^{2}$$

(9) $$MAE=\frac{1}{n}\sum_{i=1}^{n}\left|y_{i}-\overset{´}{y_{i}}\right|$$

(10) $$MBE=\frac{1}{n}\sum_{i=1}^{n}(y_{i}-\overset{´}{y_{i}})$$

Where:

${\text{y}}_{\text{i}}$ is predicted biophysical data;

$\overset{´}{{\text{y}}_{\text{i}}}$ is the observed biophysical data;

$\bar{\text{y}}$ is the average of the observed biophysical data;
n is the number of field samples used for validation.

In the validation phase, the overall forecasting accuracy was evaluated by calculating both relative deviations and absolute deviations of the predicted values from the observed values. This approach was employed to thoroughly assess the accuracy of the predictions.

## 3 Results

### 3.1 Maize plant spectrum data

**Figure 2:**
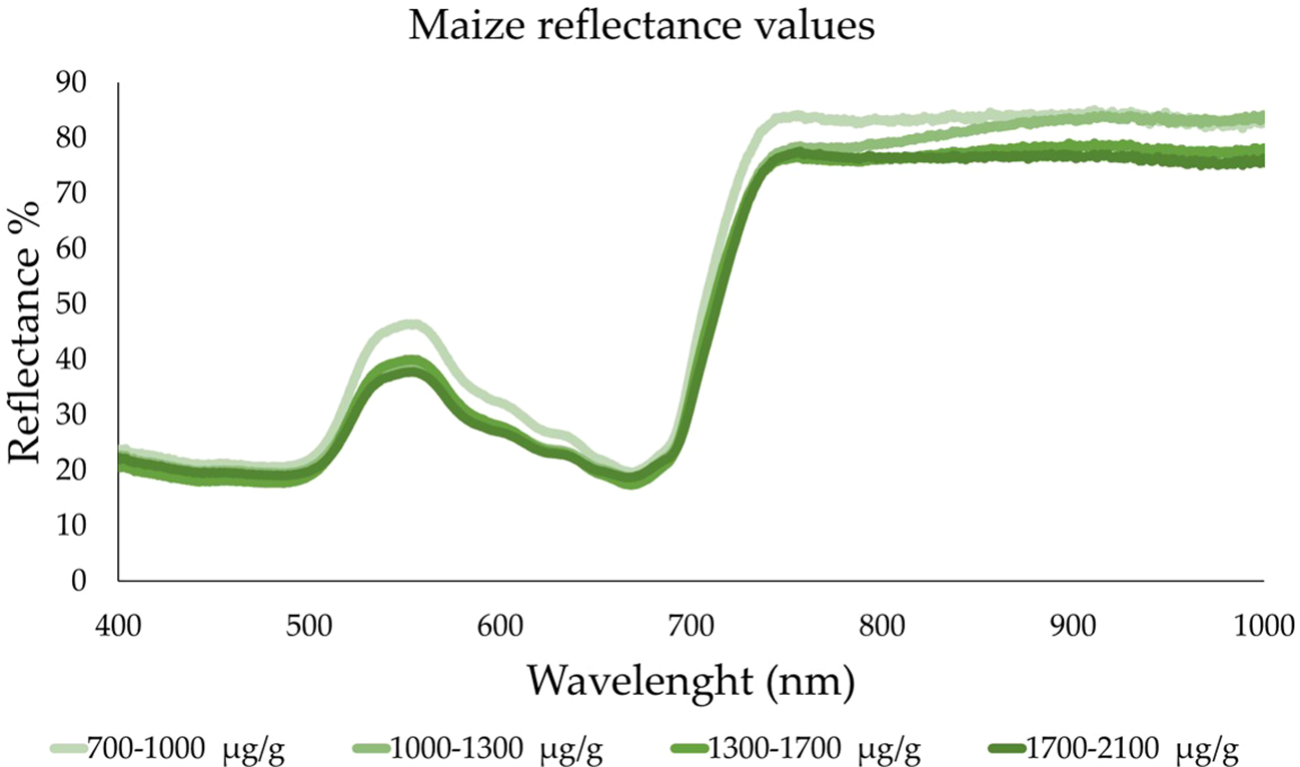
Maize plant spectrum data.

Chlorophyll reflectance profiles were evaluated across 400–1000 nm. The lowest chlorophyll content was 744 µg/g and the highest was 2,039 µg/g. Leaves with high chlorophyll content showed reflectance values between 20–25%, which increased as chlorophyll decreased. Carotenoid reflectance peaked at 520–580 nm, reaching nearly 35% at high chlorophyll content, and increased proportionally with decreasing chlorophyll. Plant stress was indicated by high reflectance in the 500–700 nm range (**Figure 2**).

### 3.2 Model building

In this section, we describe the model-building process for predicting plant health and biomass based on spectral and vegetation data. The average vegetation cover is 14.664 ± 3.131% indicating moderate variability in the data. The mean NDVI is 0.111 ± 0.033, reflecting the health and density of the vegetation. Chlorophyll and carotenoid contents, critical indicators of plant health and photosynthetic activity, have means of 1220.52± of 327.153 µg/g and 217.08 ± 53.763 µg/g, respectively, with relatively high variability. Biomass measurements show an average wet biomass of 49.573 ± 9.854 g and dry biomass of 6.992 ± 1.644 g. The dry matter content percentage averages at 0.141 ± 0.015% suggesting consistency in the dry matter content across the samples (**Table 2**). These data were used to build predictive models that link spectral information to key plant traits, allowing for more accurate estimation of biomass and pigment content across varying environmental conditions.

**Table 2:** Descriptive statistics of the biophysical parameters.

Biophysical parameters	Mean	Std. Deviation	N
Vegetation cover	14.664	3.131	48
NDVI	0.111	0.033	48
Total chlorophyll (µg/g)	1220.52	327.153	48
Total carotenoid (µg/g)	217.08	53.763	48
Wet Biomass (g)	619	124	48
Dry biomass (g)	87.4	20.5	48
Dry matter content %	0.141	0.015	48

**Figure 3:**
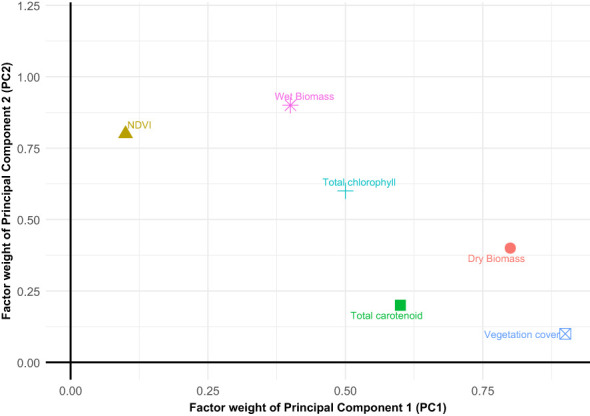
Result of principal component analysis – scatter plot of factor weights of the first component (PC1 x-axis) and the second component (PC2 y-axis) (Area ratio is the vegetation cover).

In evaluating the relationship between spectral data and biophysical parameters (**Table 2**), PCA was conducted using SPSS software (version 25). In the context of PCA, the relationships between carotenoids, chlorophyll, and NDVI are captured as principal components that represent underlying physiological processes. Chlorophyll, a major contributor to light absorption and photosynthetic efficiency, and carotenoids, which mitigate oxidative stress, often dominate the variance in spectral data. PCA enables the identification of these key patterns, linking the primary components to physiological traits such as photosynthetic capacity and stress responses, thereby enhancing the interpretation of spectral measurements The Kaiser-Meyer-Olkin (KMO) test yielded a result of 0.644, suggesting moderate suitability for principal component analysis, and the Bartlett test was found to be significant, indicating that the data was appropriate for factor extraction. The **Figure 3** describes the scatter plot of factor weights of the first component (PC1 x axis) and the second component (PC2 y axis). This PCA was conducted to test multicollinearity among the parameters, assess the variance, and depict the independent variables. According to the results, factor 1 encompassed chlorophyll, carotenoid, mean NDVI. Based on these findings through the principal component analysis, a significant correlation can be inferred between the two pigments and between the pigments and the average NDVI values. The parameters associated with factor 2 include the area ratio, wet and dry biomass properties, indicating a significant correlation between these variables as well. Factor 3 exclusively comprised dry matter content, suggesting only a very slight relationship with the other parameters (an independent variable). Nevertheless, the determination of dry matter content is a crucial aspect of crop production, highlighting the importance of its rapid and accurate measurement (**Figure 3**).

**Figure 4:**
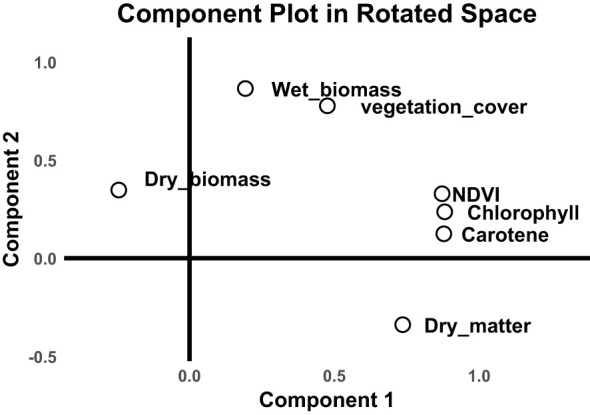
The component plot in rotated space, a result of PCA.

The component plot in **Figure 4** gives, in detail, the relationship of variables clustering together and their contribution toward the principal component. The rotated axes explain the highest variance among the variables, with variables closer together being highly correlated. Component 1 seems to be related to factors reflecting photosynthetic activity and health status of vegetation, represented by NDVI, Chlorophyll, and Carotene. Component 2 appears to emphasize factors related more toward biomass, in particular wet biomass, and vegetation cover while dry biomass is in the negative range of Component 2 (**Figure 4**). Wet biomass and vegetation cover are closely related, indicating they likely follow similar patterns in the data. NDVI, Chlorophyll, and Carotene are also strongly related, suggesting they measure similar aspects of plant properties. Dry matter occupies a unique position, isolated from other variables, suggesting it may represent an independent factor.

The regression analysis between the mean NDVI and chlorophyll content indicates that both the constant and slope significance levels are p<0.05, i.e. chlorophyll is well estimated from NDVI. Nevertheless, the presence of correlation is a fundamental prerequisite for factor analysis, as it allows variables to be consolidated into factors. A correlation matrix was computed to explore the relationships among the parameters constituting each factor, assuming a strong relationship among these parameters. The strength of the correlations among the identified parameters during factor analysis was thoroughly analyzed (**Table 3**). Vegetation cover showed a strong positive correlation with NDVI (r = 0.714, p < 0.001), confirming NDVI’s effectiveness in reflecting green canopy cover. Moderate correlations were observed with total chlorophyll (r = 0.522) and carotenoids (r = 0.406), indicating that increased vegetative cover is associated with higher pigment concentrations. Strong correlations were also found between vegetation cover and both wet biomass (r = 0.739) and dry biomass (r = 0.762), suggesting that canopy closure aligns well with biomass accumulation. The correlation with dry matter content was weaker (r = 0.300), reflecting the limited ability of cover-based indices to capture internal tissue properties such as water content. NDVI itself showed strong positive correlations with chlorophyll (r = 0.800) and carotenoids (r = 0.737), which is expected given its sensitivity to photosynthetically active pigments. NDVI also correlated moderately with wet biomass (r = 0.402) and more strongly with dry biomass (r = 0.613), underscoring its value in biomass estimation. Interestingly, a moderate correlation was found with dry matter content (r = 0.546), implying that NDVI may indirectly reflect water status to some extent. The relationship between chlorophyll and carotenoids was extremely strong (r = 0.923), highlighting their co-regulation in plant physiology. Both pigments showed weaker but still significant correlations with biomass variables and dry matter content (ranging from 0.3 to 0.47), indicating their limited standalone predictive power for structural parameters compared to NDVI. Overall, the data suggest that NDVI provides a reliable proxy for vegetation cover and biomass and offers insight into pigment content and water status, although with some limitations. For the variables of factor 1, there exists a strong positive relationship among pigment contents, a strong positive correlation between pigment contents and mean NDVI. The components of factor 2 exhibit a strong correlation encompassing the area ratio, wet biomass, dry biomass, and NDVI. The third component, representing dry matter content, demonstrates a positive but only moderate correlation with variables belonging to other factors.

**Table 3:** Correlation coefficient between the pigments and data derived from multispectral images.

Tested parameters	Statistics	NDVI	All chlorophyll	All carotenoid	Wet biomass	Dry biomass	Dry matter content
Vegetation cover	Pearson Corr.	.714^**^	.522^**^	.406^**^	.739^**^	.762^**^	.300^*^
	Sig. (2-tailed)	*.000*	.000	.004	*.000*	*.000*	.038
	N	*48*	48	48	*48*	*48*	48
NDVI	Pearson Corr.	1	.800^**^	.737^**^	.402^**^	.613^**^	.546^**^
	Sig. (2-tailed)		*.000*	*.000*	.005	*.000*	.000
	N	48	*48*	*48*	48	*48*	48
	Sig. (2-tailed)		.000	.008	.016	.062	.973
	N		48	48	48	48	48
All chlorophyll	Pearson Corr.		1	.923^**^	.324^*^	.473^**^	.400^**^
	Sig. (2-tailed)			*.000*	.025	.001	.005
	N		48	*48*	48	48	48
All carotenoid	Pearson Corr.			1	.296^*^	.488^**^	.464^**^
	Sig. (2-tailed)				.041	.000	.001
	N			48	48	48	48
Wet biomass	Pearson Corr.				1	.851^**^	.056
	Sig. (2-tailed)					*.000*	.706
	N				48	*48*	48
Dry biomass	Pearson Corr.					1	.568^**^
	Sig. (2-tailed)						.000
	N					48	48

* Significance level p <0.05.

** Significance level p <0.01.

Similar findings were observed for carotenoids. Through regression analysis between the NDVI and carotenoid content, an estimating equation was established (**Table 4**). Both the constant and slope exhibited significance levels of p < 0.05.

**Table 4:** Evaluation of linear regression analysis of chlorophyll and mean NDVI.

Model		Unstandardized Coefficients		Standardized Coefficients	t	Sig.
		B	Std. Error	Beta		
1	(Constant)	-2700	103.089		20.517	.000
	NDVI	8025.2	888.369	.800	9.034	.000
a. Dependent Variable: total chlorophyll						

A notable correlation was identified between vegetation cover and both wet biomass and dry biomass. Consequently, estimating equations were formulated for these relationships. The equation for wet biomass exhibited significance levels for both constant and slope at p < 0.05 (**Table 5**).

**Table 5:** Evaluation of linear regression analysis of carotenoid and mean NDVI.

Model		Unstandardized Coefficients		Standardized Coefficients	t	Sig.
		B	Std. Error	Beta		
1	(Constant)	-376.96	19.061		18.499	.000
	NDVI	1216	164.262	.737	7.403	.000
a. Dependent Variable: total carotenoid						

The linear regression analysis done in this study shows a strong positive linear relationship between wet biomass content and vegetation cover, as seen in **Table 6**. From the unstandardized coefficients, the constant intercepts at 193.62, which is the point where “vegetation cover” is zero. Then again, the unstandardized coefficient for “vegetation cover” is 29.053, showing that with each step that “vegetation cover” takes, the dependent variable increases by approximately 29.053 units. The Beta-value for the vegetation cover is 0.739, indicating a strong positive effect of vegetation cover on the dependent variable. Based on its t-value of 7.430 and significance level, p <.001, the effect of vegetation cover is highly significant. Overall, vegetation cover is strongly supported as a means for estimating wet biomass by this model.

**Table 6:** Evaluation of the linear regression analysis of wet biomass and vegetation cover.

Model		Unstandardized Coefficients		Standardized Coefficients	t	Sig.
		B	Std. Error	Beta		
1	(Constant)	193.62	46.88		3.304	.002
	vegetation cover	29.053	3.13	.739	7.430	.000
a. Dependent Variable: Biomass wet						

The unstandardized coefficient (B) for constant (intercept) from **Table 7** is 14.008, which gives the expected value of the dependent variable when “vegetation cover” is zero. The unstandardized coefficient for “vegetation cover” is 14.008, which means that for every one-unit increase in vegetation cover, the dependent variable increases by about 14 units. The vegetation cover standardized coefficient, Beta, is 0.762, showing that the relationship between vegetation cover and the dependent variable is strong and positive. The t-value is large, 7.992, and the p-value, p <.001, demonstrates that this effect is highly statistically significant. On the other hand, the constant was not statistically significant, p = 0.142, and hence the intercept may not be meaningful.

**Table 7:** Evaluation of linear regression analysis of dry biomass and vegetation cover.

Model		Unstandardized Coefficients		Standardized Coefficients	t	Sig.
		B	Std. Error	Beta		
1	(Constant)	14.008	7.51		1.493	.142
	vegetation cover	5.005	.50	.762	7.992	.000
a. Dependent Variable: Biomass_dry						

### 3.3 Linear models development

Linear regression (LR) is a simple and interpretable statistical method used to model the linear relationship between dependent and independent variables. In this context, the predictions are based on plant biophysical properties. Some of the key assumptions underlying LR include homoscedasticity-the variance of the training samples is constant-the training data is normally distributed and statistically independent, and there exists a linear relationship between the dependent and independent variables (Montgomery et al., 2021). The linear models were constructed using in RStudio Desktop version 2024.12.0 + 467. The model to predict carotenoid using NDVI is based on the following Equation 11:


(11) Carotenoid=−376.96+1216×NDVI

This model predicts the total carotenoid content based on the NDVI value, with an intercept of -376.96 and a slope of 1216. This model was derived using the unstandardized coefficients from the regression analysis, with the results indicating a strong relationship between NDVI and carotenoid content. The statistical significance of the model was confirmed through t-tests and p-values (**Table 5**).

The linear regression model to predict chlorophyll based on NDVI is Equation 12:


(12) Chlorophyll=−2700+8025.2×NDVI

This model predicts the total chlorophyll content based on the NDVI value, with an intercept of -2700 and a slope of 8025.2. The model is statistically significant, as indicated by the t-value (9.034) and p-value (0.000) (**Table 4**).

The linear regression model to predict dry biomass based on vegetation cover is Equation 13:


(13) Dry Biomass=14.008+5.005×Vegetation Cover

This model predicts the dry biomass based on vegetation cover, with an intercept of 14.008 and a slope of 5.005. Although the model’s coefficient for vegetation cover is statistically significant (p-value = 0.000), the intercept’s p-value (0.142) suggests it is not significant at the typical 0.05 threshold (**Table 7**). Nevertheless, the model offers valuable insights into the relationship between vegetation cover and dry biomass.

The linear regression model to predict wet biomass based on vegetation cover is Equation 14:


(14) Wet Biomass=193.62+29.053×Vegetation Cover

This model predicts the wet biomass based on vegetation cover, with an intercept of 193.62 and a slope of 29.053. The model is statistically significant, as shown by the t-value (7.430) and p-value (< 0.001), with regression analysis conducted and evaluated in RStudio.

### 3.4 Validation

**Figure 5:**
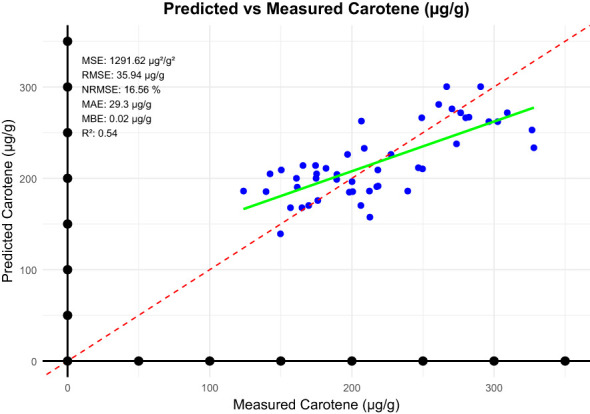
Statistical metrics between predicted carotenoid and measured carotenoid.

In our study, we developed a linear model to predict carotenoid based on NDVI, evaluating its performance through several key metrics from equation 5 to 11 The MBE was calculated at -0.019 µg/g, indicating a slight underestimation of carotenoid values. The MSE stood at 1291.62 µg/g, reflecting the overall prediction error of the model. Furthermore, the MAE was found to be 29.30 µg/g, representing the average magnitude of error in our predictions. The RMSE was calculated at 35.94 µg/g, underscoring the model’s predictive accuracy. However, the NRMSE reached 17%, suggesting a significant level of reliability in the predictions relative to the observed range of carotenoid values (**Figure 5**). These results highlight both the strengths and limitations of the model, providing valuable insights for future research in this area. This relatively low prediction accuracy can be attributed to several factors such as Limited Spectral Information: NDVI is derived from a narrow spectral range, which may not fully capture the complexity of carotenoid variability. Simplistic linear modelling approach may fail to capture non-linear relationships between NDVI and carotenoid concentrations, especially under varying environmental and physiological conditions. To improve model performance, future research should consider incorporating additional spectral bands, such as green-edge and red-edge indices, which are more sensitive to carotenoid and chlorophyll variations. Additionally, exploring non-linear modelling approaches, such as machine learning techniques, could better capture the intricate relationships between spectral indices and carotenoid levels.

**Figure 6:**
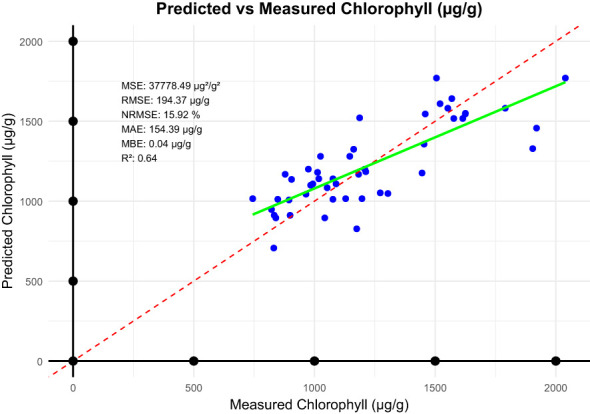
Statistical metrics between predicted chlorophyll and measured chlorophyll.

Model performance for the linear prediction of chlorophyll level using NDVI was evaluated based on some key error metrics, as shown in **Figure 6**. The MBE was -0.1962 µg/g, hence there is a trend to underestimate the chlorophyll levels. The overall prediction error was high, considering an MSE of 37778.53 µg/g. For the MAE, the estimated value was 154.4062 µg/g; this represents the actual level of deviation from the observed data. The RMSE was 194.34 µg/g, which further points to how the model had some predictability challenges. Notably, the NRMSE reached 15.00%, therefore meaning that a very high level of reliability of the predictions with respect to the range of chlorophyll values. From these, it can be seen how both the potential and limitations of the model point to possible future research avenues. The high prediction errors likely stem from such as environmental variability. Factors such as lighting conditions and canopy structure may have introduced noise into the NDVI measurements. Model simplicity such as the linear model may not adequately account for the complex interactions between chlorophyll content and NDVI under varying growth conditions. Improving chlorophyll prediction could involve incorporating additional vegetation indices that leverage green-edge and near-infrared bands, which are more closely associated with chlorophyll absorption. Employing advanced modeling techniques, such as random forests or neural networks, could also help capture the non-linear dynamics affecting chlorophyll variability.

**Figure 7:**
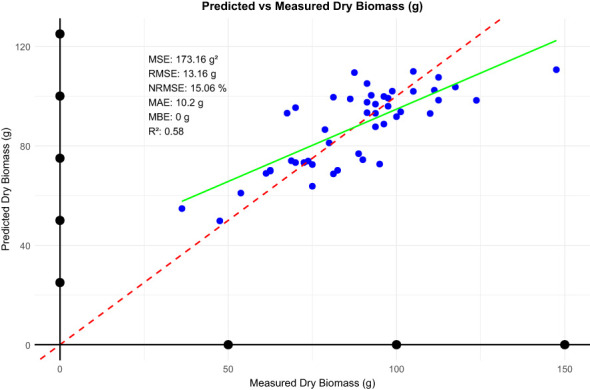
Statistical metrics between predicted and measured dry biomass.

In our analysis, the linear model performance using vegetation cover to estimate dry biomass is promising in terms of accuracy and reliability (**Figure 7**). The MBE of -0.00099 g indicates negligible bias, suggesting the model’s predictions are effectively unbiased. The MAE of 10.19 g and RMSE of 13.16 g indicated a moderate level of prediction error; RMSE was slightly higher, as it is sensitive to large deviations. Notably, an NRMSE of 15.06 reflects that the model has a strong predictive power, since it involves only a small fraction of data variability. These metrics in total give evidence of the potential for the linear model to be useful and reliable for estimating the dry biomass by vegetation cover, suitable for ecological and agricultural applications. However, the model’s limitations include Simplified Assumptions: Linear models may oversimplify the relationship between vegetation cover and biomass, especially in heterogeneous field conditions. Spectral Saturation: At high vegetation densities, NDVI and related indices may saturate, reducing sensitivity to further biomass increases. To address these issues, future efforts could focus on integrating texture-based features or structural indices to complement vegetation cover measurements. Additionally, using advanced regression techniques, such as support vector machines or gradient boosting, may enhance the model’s robustness.

**Figure 8:**
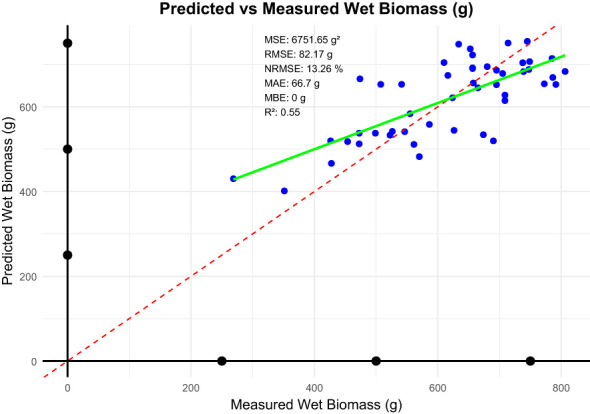
Statistical metrics between measured and predicted wet biomass (%).

Here in, the linear model (**Figure 8**) for prediction of wet biomass from vegetation cover (area ratio) has low bias, as indicated by near-zero MBE and no systematic over- or under-predictions. For an MAE of about 66.7 g and a typical prediction deviation shown by the RMSE about 82.2 g, the NRMSE is 13.26%, indicating that on average, the errors in this model are small, relative to the scale of the target variable, tending to be reasonably good predictions. Overall, the model performs rather consistently, and errors stay within a rather acceptable range for agricultural applications. Despite the acceptable performance, limitations persist such as environmental influences, that is variations in soil moisture, canopy structure, and lighting conditions may affect the reliability of vegetation cover as a predictor. Model generalizability, that is, the linear model may not generalize well across diverse environmental and crop management scenarios. Future improvements could involve incorporating additional spectral and environmental variables, such as soil moisture indices or weather data, to enhance model accuracy. Exploring hybrid models that combine linear and non-linear components could also improve predictions by capturing complex interactions more effectively.

## 4 Discussion

This study presents a leap in precision agriculture using ground-based spectral imaging for real-time monitoring of biomass in maize under open-field conditions. It proposes a low-cost and scalable approach compared to traditional methods and tackles challenges that are usually involved with remote sensing technologies that often rely on UAVs or satellites. Ground-based systems, by providing direct, proximal measurements, reduce dependence on environmental conditions and logistical constraints, thus making hyperspectral imaging more feasible for a wide range of agricultural operations, especially in resource-poor conditions.

Our study demonstrates the potential of hyperspectral imaging combined with linear regression modelling for estimating wet and dry biomass, chlorophyll, and carotenoid content in maize. The predictive performance of our linear regression models was moderate, with R² values ranging from 0.54 to 0.64. Notably, the chlorophyll model achieved an R² of 0.64 and an NRMSE of 15%, outperforming some machine learning approaches based on SPAD values (e.g (Guo et al., 2022): R² = 0.462–0.570, MAE = 0.886–1.739 µg/g). However, our results did not reach the highest accuracies reported in the literature, such as those from UAV-based hyperspectral imaging and advanced machine learning, which have achieved R² values above 0.9 and NRMSE as low as 8.98% (Yang et al., 2023). This highlights a key trade-off: while advanced models and platforms offer higher accuracy, our approach remains accessible and computationally efficient, making it suitable for routine crop monitoring and resource-limited settings.

Our chlorophyll estimation model (R² = 0.64, NRMSE = 15%) is competitive compared to UAV-based approaches. For example Li et al. (2022), reported an R² of 0.75 and RMSE of 2.09 mg/L using UAV multispectral data and machine learning, but their workflow required complex vegetation index (VI) optimization under varying canopy coverage. In contrast, our simpler linear regression framework achieves moderate accuracy with lower computational demands. The statistical significance of NDVI for chlorophyll estimation is well supported, as shown by Miraglio et al. (2019), who reported strong correlations between NDVI and various spectral bands and indices (e.g., NDVI: NDBR r = 0.952, NDVI: NDWI r = 0.977). Our results reinforce NDVI’s sensitivity to leaf chlorophyll content, confirming its utility in precision agriculture.

For biomass estimation, our models for wet and dry biomass (R² = 0.58 and 0.55, respectively) are somewhat lower than those achieved by UAV-based hyperspectral imagery and advanced ML methods (Yang et al., 2022: R² = 0.81, RMSE = 0.27 t/ha). These higher-performing models benefit from the ability to calculate numerous narrowband VIs and capture subtle spectral variations, particularly during late growth stages. However, when compared to the NDVI-based maize above-ground biomass (AGB) model by Meiyan et al. (2022), which achieved R² = 0.79 and NRMSE = 31.79%, our wet biomass model demonstrated superior accuracy (RMSE = 13.16 g, NRMSE = 13.26%). This may be due to the enhanced reliability of our imaging sensor in controlled conditions, suggesting that proximal sensing can deliver robust results with broader applicability, even if it does not match the highest precision of UAV-based systems.

Our biomass prediction performance is also consistent with satellite-based studies (Madundo et al., 2023: R² = 0.60–0.74), highlighting the trade-off between broader applicability and slightly reduced precision compared to UAV-specific models. The broader applicability of our sensor technology potentially reduces variability in biomass estimation and increases robustness under diverse field conditions. While our models do not match the performance of hyperspectral and ML-based approaches, they provide a simpler and cost-effective alternative with competitive accuracy in specific applications. Future work could explore integrating narrowband VIs or adopting advanced ML algorithms to further improve the predictive power of our models.

For carotenoid estimation, our model exhibited higher RMSE and variability compared to NDVI-based approaches using hyperspectral data (Miraglio et al., 2019): RMSE = 1.34 μg/cm²). This discrepancy is likely due to the heterogeneous distribution and greater spectral overlap of carotenoids, as well as their sensitivity to short-term stress, which complicates their isolation in multispectral datasets (Berger et al., 2022). The superior performance reported by (Sonobe and Hirono, 2023) may be attributed to their use of hyperspectral imagery, which provides greater spectral resolution and enhances the sensitivity of NDVI to carotenoid variations. This aligns with the broader trend in precision agriculture, where hyperspectral data often outperform multispectral systems in capturing fine-scale biochemical changes. However, the trade-offs in cost, accessibility, and computational requirements make our approach using simpler imaging systems more feasible for widespread adoption, especially in resource-limited settings.

Our results reaffirm NDVI’s robustness for LAI estimations and carotenoid prediction across different contexts. The implications for precision agriculture are significant: NDVI-based models, even when not optimized with hyperspectral data, can still provide actionable insights for crop monitoring, nutrient management, and yield optimization. This is particularly valuable for large-scale or economically constrained farming operations where rapid and cost-effective assessments are prioritized.

A comparative analysis of experimental designs across studies reveals that structured methodologies, such as nested and blocking designs, are essential for accurately estimating spatial heterogeneity of biophysical parameters. For example, Nigon et al. (2014) used a nested design to investigate nitrogen sufficiency indices in potatoes, allowing for hierarchical dependencies in field variability. Similarly, Morier et al. (2015) employed a randomized complete block design to reduce environmental gradient effects on hyperspectral vegetation indices, yielding robust predictions. Our study follows these best practices by employing a spatially heterogeneous sampling strategy within an irrigated maize plot, combined with stratification by phenological stage using the BBCH scale. This design mirrors the nested framework in its hierarchical structure and the blocking approach in its temporal segmentation, facilitating nuanced correlations between NDVI, pigment content, and biomass parameters.

One limitation of our study is the use of only two maize varieties within a narrow spatio-temporal extent, which may constrain the generalizability of our findings. This concern is echoed by Sims and Gamon (2002), who highlighted how varietal differences in leaf traits can influence spectral responses, and Mróz et al. (2024), who emphasized the importance of validating spectral models across diverse environments.

Recent studies have demonstrated that machine learning models, such as random forests and support vector regression, can outperform linear models under similar field conditions, offering improved accuracy in biomass estimation (Zhou et al., 2025). For example, Liu et al. (2022) combined partial least squares regression (PLSR) and Gaussian process regression (GPR) for potato AGB estimation, achieving R² values up to 0.72 and NRMSE as low as 15.04%. In comparison, our results are similar in magnitude, suggesting that linear models, when paired with hyperspectral data, remain a viable option for specific modelling tasks.

Despite the high accuracy achieved in biomass estimation, our findings emphasize the influence of environmental conditions, sensor calibration, and data preprocessing on hyperspectral imaging performance. Future research should explore the integration of hyperspectral data with complementary remote-sensing techniques, such as LiDAR or thermal imaging, to create more robust and scalable AGB estimation models applicable across diverse crops and field conditions. Additionally, incorporating vegetation indices with texture analysis could help mitigate noise and background interference, addressing the saturation issues associated with high biomass levels and further improving model efficiency.

These models can enhance precision agriculture tools through three key pathways. Smart irrigation scheduling benefits from integrating chlorophyll maps with soil moisture data in decision-support platforms, enabling dynamic water allocation through AI-driven feedback loops that optimize resource use (Bhattacharya and Pandey, 2023: Alvim et al., 2022 and Murugan et al., 2016). Nitrogen management leverages temporal vegetation index trends from UAV/satellite fusion to trigger site-specific fertilizer applications during critical growth stages, mirroring workflows that reduced nitrogen over-application by 18% in maize via simulation-model integration (Qiao et al., 2022; Chen et al., 2023). Yield optimization frameworks could automate data assimilation by combining canopy distribution models with irrigation controllers, balancing vegetative and reproductive growth phases using biomass predictions (Bwambale et al., 2024). This integration aligns with emerging frameworks that fuse multisource remote sensing data to improve crop monitoring and resource efficiency (Zhu et al., 2021; Ma et al., 2022). It also underscores critical areas for refinement, such as optimal band selection and multimodal data fusion, to enhance the accuracy and applicability of AGB monitoring systems.

In summary, our study highlights the utility of hyperspectral imaging and linear regression for estimating key biophysical parameters in maize. While our models do not match the highest-performing machine learning or UAV-based methods, they offer a competitive, accessible alternative for biomass and pigment estimation. The simplicity and interpretability of linear models, combined with robust experimental design, ensure broad applicability in both research and practical agricultural monitoring. Future research should focus on integrating advanced algorithms and multimodal data sources to further enhance predictive accuracy and operational scalability.

## 5 Conclusion

This study demonstrates that vegetation cover data obtained from the ADC multispectral camera can effectively estimate both wet and dry biomass in maize, as well as chlorophyll and carotenoid content. NDVI-based estimations achieved net errors of less than 15% for chlorophyll and 17% for carotenoids, while biomass predictions showed errors of 13% and 15% for wet and dry biomass, respectively. These results highlight the potential of multispectral imaging as a practical tool for precision agriculture, particularly in irrigation management and nutrient monitoring. However, several challenges remain. The current methodology is labor-intensive, offers limited area coverage per deployment, and lacks automation in data acquisition and processing—factors that hinder large-scale implementation.

To address these limitations, future work should focus on mounting multispectral sensors on autonomous ground vehicles (AGVs) to reduce labor demands and extend spatial coverage. Integrating edge-computing systems for real-time data analytics would further streamline workflows and enable faster decision-making in the field. Additionally, validating models across multi-location trials and diverse agroecological zones is essential to enhance generalizability and ensure reliable performance under varied conditions. Expanding the modelling framework to include diverse maize varieties and incorporating additional biophysical indicators—such as leaf area index and pigment profiles—can improve the robustness and accuracy of biomass and chlorophyll predictions. These advancements will be critical for developing scalable, efficient, and automated monitoring systems to support sustainable crop production and resource use in modern agriculture.

## Data Availability

The original contributions presented in the study are included in the article/Supplementary Material. Further inquiries can be directed to the corresponding author.
